# Dental Education in Hungary

**Published:** 1880-11

**Authors:** Jos. Arkovy

**Affiliations:** Dr. of Med. and Surg. Master of Dent.


### DENTAL EDUCATION IN HUNGARY.

BY JOS. ARKOVY, DR. OF MED. AND SURG. MASTER OF DENT.

In Hungary, as well as in the politically and materially connected Austria, a special dental education does not exist. Neither Hungary nor Austria possesses such a distinguished institution as, for example, the Dental Hospital of London, and the London School of Dental Surgery; one consequence of that is, that dental education is taken up in the course of the general medical education, through which it has all right to rank with, and be as respected as, any other specialty.

There are in Hungary three universities, which are Government Institutions—that is, they are high schools, which are inspected by Government authorities, conducted in the highest manner, and with the rights of administration of the interior affairs secured to the faculties. One of the oldest in Europe is the university of Budapest; those of Kolozsvar and of Zagrab have been founded but a few years. In Zagrab there is not, as yet, any medical faculty. Budapest is the only university which at present possesses two chairs for dentistry. The one confers the title of professor, the other of lecturer. This last office is now vacant. In former years, to meet the need felt for medical men in the country, a course of education existed for so-called licentiates in surgery; this provisorious system was abolished about nine years ago. The licentiates at that time were permitted to practice dentistry, but since then only those who are doctors of medicine are admitted to the dental examinations.

In giving the legitimate way of acquiring a diploma as dentist, I will keep in mind the regulations respecting the education and examination of candidates in Great Britain.

The preliminary general education in Hungary, where education is compulsory, is obtained partly through four elementary classes, and partly through the eight classes of the gymnasium. Pupils are received into the former when five or six years old, and into the latter from nine to ten years old. No age is named at which a pupil may go to the universities. The student leaves the gymnasium when he has passed what is called the examination of maturity, which comprises all branches of learning. The testimonial gives him a right to enter the university. In order to spare the reader the particularizing of every branch of education, I will only mention that a scholar in the fourth or fifth class is quite equal to one who can pass an English preliminary examination. The student at the university, during five years, has to be present at all the medical lectures, and to make practical exercises similar to the requirements of English licensing bodies, before he is admitted to the primary examination, and after an interval of several months he is admitted to the pass-examination. Four years ago a new system of examination was introduced, according to which the former two examinations were divided into severally oral, written, and practical divisions; and special branches, as surgery, midwifery, opthalmology, were included. Only after all these examinations does the candidate obtain the diploma of a Doctor Universæ Medicinæ.

The special lectures in dentistry embrace to the fullest extent the anatomy of the mouth—embryology, physiology, pathology, surgery, demonstrations on some models, and different subjects out of the odontotechnic. The special examination is oral, and consists principally of questions relating to the teeth, mouth and adjoining parts.

The university, as also the great town's hospital, has no separate clinical departments for teeth and mouth diseases, and therefore the young dentist is obliged to acquire practical knowledge during the years before or after his studies in the technical and operating places of some celebrated dentist. Still, this last method of acquiring practical knowledge in dentistry was not required to secure a diploma, as the university had only to watch over the examinations of scientific knowledge. The only requisite necessary to pass an examination in dentistry was to have attended during one session the lectures of the professor of dentistry. *Now*, everybody may, or may not visit these, quite as the student pleases.

From what has already been said, it is evident that not only is there no pretention in Hungary towards a special school for dentistry, but that any one who desires to follow this profession must get the practical knowledge, or rather necessary experience in some manner, apart from the university which only imparts scientific knowledge.

What has just been written upon a dentist's education in Hungary can also be said of Austria.

In respect of practice, the same may be said of the whole monarchy of Austro-Hungary. But on the other hand, in Hungary, according to Government laws, no one may practice with a foreign diploma unless such practitioner receive special admission to practice by passing a proper examination before the medical faculty. The number of dentists in Hungary is very small, for in the capital, with about 400,000 inhabitants, there are at present, only eleven. Here, as everywhere else, unqualified persons exist, and practice on the public. A few years ago there was a proposition moved by the medical faculty to the Minister of Public Education to set on foot a special School for Dentists, but, fortunately, nothing has yet been done. It would be more advantageous for the profession should this idea not be carried out, as it proposes to allow licentiates in dentistry, *only*, to practice, persons not of necessity possessing scientific knowledge, which, for Hungary, could not be regarded as a step towards progression.

Every Doctor of Medicine who passed his examinations in the old system had the right either to go through the examination for a dental diploma, which gave him the title of Master of Dentistry, or he had also the full right to choose dentistry for his special line without any dental diploma; only on the basis of being an M. D. Since the new system of examination became general, the title of Doctor Universæ Medicinæ is considered to imply also dentistry, as well as otology, laryngology, or any branches of medicine and surgery. In consequence of that new system all kinds of special diplomas ceased to exist.—*British Monthly Review of Dental Surgery.*

